# Differential Post-Surgical Metastasis and Survival in SCID, NOD-SCID and NOD-SCID-IL-2Rγ^null^ Mice with Parental and Subline Variants of Human Breast Cancer: Implications for Host Defense Mechanisms Regulating Metastasis

**DOI:** 10.1371/journal.pone.0071270

**Published:** 2013-08-14

**Authors:** Chloe C. Milsom, Christina R. Lee, Christina Hackl, Shan Man, Robert S. Kerbel

**Affiliations:** Department of Medical Biophysics, University of Toronto and Biological Sciences Platform, Sunnybrook Research Institute, Toronto, Ontario, Canada; University of Alabama at Birmingham, United States of America

## Abstract

We compare for the first time, the metastatic aggressiveness of the parental MDA-MB-231 breast cancer cell line and two luciferase-tagged *in vivo*-derived and selected pro-metastatic variants (LM2-4/luc^+^ and 164/8-1B/luc^+^) in SCID, NOD-SCID and NOD-SCID-IL-2Rγ^null^ (NSG) mice following orthotopic implantation and primary tumour resection. The variants are known to be more aggressively metastatic in SCID mice, compared to the parental line which has limited spontaneous metastatic competence in these mice. When 2×10^6^ cells were injected into the mammary fat pad, the growth of the resultant primary tumours was identical for the various cell lines in the three strains of mice. However, metastatic spread of all three cell lines, including the MDA-MB-231 parental cell line, was strikingly more aggressive in the highly immunocompromised NSG mice compared to both NOD-SCID and SCID mice, resulting in extensive multi-organ metastases and a significant reduction in overall survival. While these studies were facilitated by monitoring post-surgical spontaneous metastases using whole body bioluminescence imaging, we observed that the luciferase-tagged parental line showed altered growth and diminished metastatic properties compared to its untagged counterpart. Our results are the first to show that host immunity can have a profound impact on the spread of *spontaneous* visceral metastases and survival following resection of a primary tumour in circumstances where the growth of primary tumours is not similarly affected; as such they highlight the importance of immunity in the metastatic process, and by extension, suggest certain therapeutic strategies that may have a significant impact on reducing metastasis.

## Introduction

Our laboratory has developed a series of models of advanced metastatic disease for experimental therapeutic studies [1]. These models involve variants of human tumour cell lines grown in severe combined immune deficient (SCID) mice, including breast carcinoma [2], [3], malignant melanoma [4], [5] and colorectal carcinoma [6], which give rise to ‘spontaneous’ metastases in multiple organ sites after resection of the primary orthotopic tumour. Primary tumour resection, a clinically common procedure, is necessary to allow the mice to survive long enough for extensive macroscopic metastases to become established, at which point systemic therapy is initiated. The rationale for this experimental approach is to duplicate more closely the treatment of patients with advanced metastatic disease who are typically enrolled in most clinical trials, especially at the phase I and II levels, and who are generally much more difficult to treat successfully. It is hypothesized that the outcomes from such preclinical studies will provide results with greater clinical predictive potential when compared to conventional models involving the treatment of a primary and often subcutaneous, transplanted tumour [1], [7].

With respect to models, we have developed a metastatic variant subline of the human breast cancer cell line MDA-MB-231, called LM2-4, which gives rise to extensive systemic (visceral) metastases in multiple sites such as the lungs, liver and lymph nodes within one month after resection of the primary orthotopic tumour in SCID mice [2], compared to less extensive metastasis arising after 3–6 months for the parental line. As a result, the parental MDA-MB-231 cell line is considered to be poorly (spontaneously) metastatic when compared to the lung metastatic variant (LM2-4), which was derived by serial *in vivo* selections of lung metastases that emerged following resection of the primary orthotopic tumour [2], [3]. In subsequent studies involving the LM2-4 variant, we found a doublet oral low-dose metronomic chemotherapy regimen consisting of daily cyclophosphamide and UFT (tegafur plus uracil, an oral 5-FU prodrug) had potent anti-metastatic effects when administered on a daily chronic basis without any breaks [2]. However, in some instances, the prolonged survival brought about by successful treatment of mice with visceral metastatic disease eventually resulted in the emergence of spontaneous macroscopic brain metastases, thus mimicking a clinical treatment phenomenon observed in breast cancer patients of growing magnitude and importance [1], [2], [4]. In this manner, we were able to obtain a variant of the LM2-4 cell line with enhanced metastatic affinity for the brain, called 164/8-1B, following long term combined treatment with cyclophosphamide and UFT.

To date, our studies have primarily utilized SCID mice, which exhibit both B-cell and T-cell deficiencies. However, it has been shown that human tumours sometimes grow more aggressively in non-obese diabetic (NOD)-SCID mice compared to SCID mice, and in turn, usually more aggressively in SCID mice compared to ‘athymic’ nude mice [8]–[12]. In addition to B- and T-cell deficiencies, NOD-SCID mice have reduced macrophage and natural killer (NK) cell function and an absence of complement activity (innate immunity), whereas nude mice display a T cell deficiency [9]–[11]. Moreover, highly immunodeficient NOD-SCID-*IL-2R*γnull (NOG - Taconic or NSG - Jackson Laboratories) mice lack the common cytokine-receptor γ-chain (IL-2Rγ), which is shared by multiple interleukin receptors and not only leads to impaired NK cell development, but also reduced dendritic cell function and macrophage activity, as well as B-cell, T-cell and innate immune deficiencies. These mice provide a superior xenotransplantation system for the successful engraftment of human cancer cells [12], [13] and appear even more ‘susceptible’ to metastasis when used as recipients for human tumour xenografts, when compared to NOD-SCID mice [8], [10]–[12], [14]–[16]. However, the majority of metastasis studies in NOG/NSG mice have involved either intravenous [9] or intra-splenic [15], [16] injection of tumour cells to form ‘artificial’ or ‘experimental’ metastases.

To our knowledge, the studies we report here represent the first attempt at comparing spontaneous metastasis following surgical resection of a primary tumour grown in SCID, NOD-SCID and NSG/NOG mice. Given the metastatic variant sublines we have previously selected and characterized, we asked whether the relative marked difference in metastatic aggressiveness between the parental MDA-MB-231 cell line, the visceral metastatic variant (LM2-4) and brain metastatic variant (164/8-1B) would be maintained when grown in NSG/NOG mice. More specifically, would the MDA-MB-231 parental line retain a slow low-grade spontaneous metastatic phenotype? Finally, would the LM2-4 and 164/8-1B variants behave even more aggressively such that metastases are commonly detected in sites such as bone and brain?

## Materials and Methods

### Ethics Statement

All animal procedures, including maintenance and determination of experimental endpoints, were performed in strict accordance with the guidelines of the Sunnybrook Research Institute Animal Care Committee and the Canadian Council on Animal Care. The protocol was approved by the Animal Research Ethics Committee at Sunnybrook Research Institute (Permit number 10–339).

### Cell Lines

Variants of the MDA-MB-231 human breast cancer cell line, LM2-4 and 164/8-1B, which were selected for a highly aggressive ability to spontaneously metastasize from an orthotopic primary tumour transplant, have been described previously [1], [2]. The LM2-4 metastatic variant subline was obtained by *in vivo* serial selections of lung metastases which emerged after orthotopic (i.e. intra-mammary fat pad) injection of the MDA-MB-231 parental line and subsequent resection of the resultant primary tumour. The spontaneous lung metastases that arose 3–6 months after primary tumour resection were then adapted to culture before being re-implanted for a second round of *in vivo* selection. The resultant cell line gave rise to visceral metastasis within 1 month after resection of a primary orthotopic tumour, and was designated LM2-4 [2]. The 164/8-1B cell line is a variant of the LM2-4 cell line with enhanced metastatic affinity for the brain. It was isolated from a spontaneous macroscopic brain metastasis that arose following the prolonged survival of mice brought about by the effective control of advanced systemic metastatic disease with a combined daily treatment of metronomic chemotherapy using oral cyclophosphamide and UFT (tegafur plus uracil, a 5-FU prodrug). The isolated brain metastasis was adapted to cell culture and designated 164/8-1B [2].

MDA-MB-231, LM2-4 and 164/8-1B cell lines were transfected with the firefly luciferase vector [17] to generate the 231P/luc^+^, *LM2-4/luc^+^ and the 164/8-1B/luc^+^ cells lines respectively. The 231P/luc^+^ line is a pooled population of clones with similar *in vitro* luciferase intensity to the LM2-4/luc^+^line. Cells were cultured in RPMI 1640 supplemented with 5% foetal bovine serum (Invitrogen Life Technologies, Inc., Burlington, ON). Cell lines were authenticated by genotyping using an Illumina mouse linkage panel and confirmed to be human in origin. *alternatively referenced as 231/LM2-4/luc+14 [3].

### Mice

Fox Chase CB17-SCID mice were purchased from Charles River (Wilmington, MA); NOD-SCID (JAX®Mice Database # 001303) and NOD-SCID-IL-2Rγnull (NSG; JAX®Mice Database # 005557) mice were purchased from Jackson Laboratories (Bar Harbor, ME). Breast cancer cells (2×10^6^) were implanted orthotopically into the right inguinal mammary fat pad of 6–8 week old female mice. Tumours were resected once average tumour volumes reached 400 mm^3^. Surgeries were performed under anaesthesia and mice were administered analgesics post-operatively to minimize discomfort. For all studies, 8 mice per group were used.

### Bioluminescence Imaging

For bioluminescence imaging following tumour resection, mice were intraperitoneally injected with 150 mg/kg of luciferin (Caliper Life Sciences, MA), 10 minutes prior to being imaged under anaesthesia using an IVIS200 system (Caliper Life Sciences) [17].

### Necropsy

Mice were sacrificed at endpoint, in accordance with institutional animal care guidelines. Macroscopic inspection of organs and tissues were performed and metastatic burden was recorded.

### Statistical Analysis

Results were reported as mean ± standard deviation (SD). Statistical significance was assessed by one-way analysis of variance (ANOVA). Kaplan-Meier survival curves were generated and the significance assessed by Log-rank tests. Statistics were generated using GraphPad Prism (GraphPad Software version 4.0, San Diego, CA). *P*<0.05 was used as the threshold of statistical significance.

## Results

### Analysis of Primary Tumour Growth

**Figure 1:**
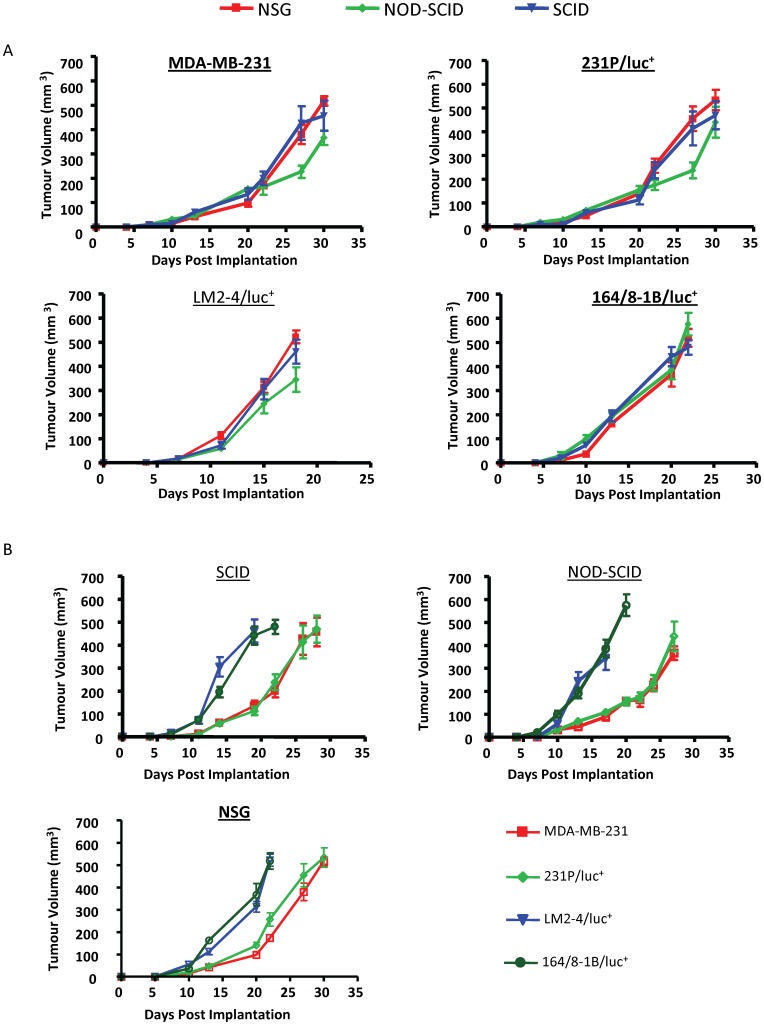
Primary tumour growth of human breast cancer cell lines in different immune-deficient mice. Two million human breast cancer MDA-MB-231 parental or luciferase-tagged parental cells (231P/luc^+^), or the luciferase-tagged lung (LM2-4/luc^+^) and brain (164/8-1B/luc^+^) metastatic variants, were injected into the right inguinal mammary fat pad of female SCID, NOD-SCID or NSG mice. Tumour growth was monitored twice weekly and plotted as tumour volume against time. (A) Comparison of primary tumour growth rate in the various strains of immunocompromised mice. (B) Comparison of the different cell lines’ growth within the three strains of immunocompromised mice. Each group contained 8 mice. Statistical analysis was assessed by ANOVA. Significance was set at P<0.05. There was no statistical difference between any of the tumour growth curves.

Orthotopic primary tumour growth was similar in SCID, NOD-SCID and NSG mice when two million cells were implanted into the mammary fat pads of female mice (Figure 1A). This is not surprising given that the number of cells injected was well above the threshold (minimum) number of cells required for tumour growth initiation [18], [19], thereby masking any potential difference in tumour growth initiation between the strains. Several studies have demonstrated an increased tumour take rate in NSG mice requiring far fewer cells than the number normally required for subcutaneous tumour growth [8] or experimental metastasis initiation [16] in NOD-SCID and SCID mice. Tumour growth initiation (tumour take) is often considered to be a reflection of tumour cell aggressiveness, particularly when limiting dilutions of tumour cells are injected to determine the threshold number of cells required for tumour growth. Under these conditions, it is plausible that tumour initiation may reflect more closely the metastatic potential of cells since the early stages of metastases require seeding of a small cell number and subsequent establishment of a distant tumour mass. However, for the purposes of our study, we chose to inject a greater number of cells in order to generate efficient tumour take in 100% of the mice implanted within a reasonable time frame, thus allowing us to assess the differences in metastatic properties in each mouse strain following primary tumour resection. Since the tumours generated from each cell line reached similar volumes within a similar length of time across the different strains of mice, we asked whether there might be major changes in the metastatic aggressiveness between the stains in a circumstance where there are no (obvious) changes in primary tumour take rates or growth.

In our experimental models, it is highly probable that the metastases arise from the primary tumour rather than being the result of spontaneous secondary lesions and so the fact that all mice had primary tumours of a similar size and for a similar time frame is of particular importance when considering factors that govern the metastatic process for two main reasons. First, if tumours become more aggressive over time in response to selection pressures within a growing tumour, tumour cells acquiring more aggressive motile characteristics would break away from the primary tumour, enter the circulation and eventually form secondary outgrowths. This would suggest that there is an increased likelihood for cells to change and metastasize the longer the growing tumour is subjected to these *in vivo* selection pressures [20], [21]. Alternatively, if tumour cells are continually being shed from the primary tumour in to the circulation, it would stand to reason that the longer the primary tumour is present, the higher the number of cells that can be shed thereby increasing their chances to successfully seed and form secondary outgrowths, leading to an overall increase in metastasis [22], [23].

When comparing the growth of the various cell lines within the different strains of mice, there is a clear separation in the tumour growth curves between the parental cell line (MDA-MB-231, whether luciferase-tagged or untagged) and the two more aggressive metastatic variants (LM2-4/luc^+^ and 164/8-1B/luc^+^) (Figure 1B). Both metastatic variants grew faster (though not significantly) than the parental lines in all three strains, though the separation is far more distinct in NOD-SCID and SCID mice than it is in NSG mice. Luciferase tagging does not appear to alter the properties of the parental cell line, insofar as primary tumour growth is concerned, and when a high number of cells are injected, since the luciferase-tagged parental cell line shows an almost identical tumour growth pattern as the untagged parental cell line in all three mouse strains (Figure 1A and B).

### Variation of Tumour Growth and the Effect of Luciferase Tagging on Tumour Variation

**Figure 2:**
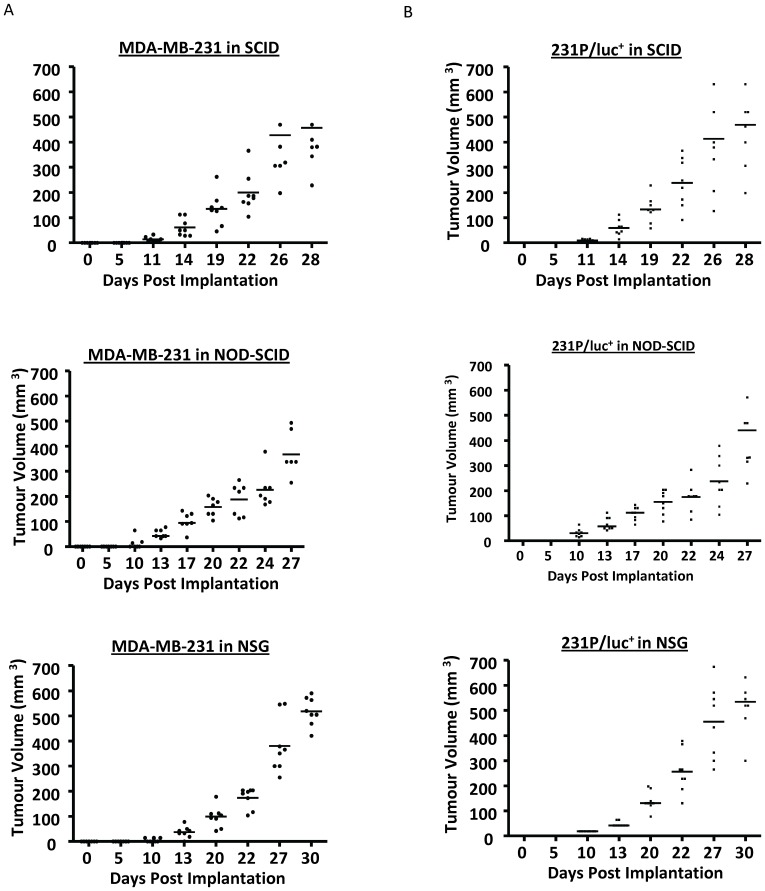
Variation in primary tumour growth in the different strains of immune-deficient mice. The parental MDA-MB-231 (A) or the luciferase-tagged parental cell line (231P/luc^+^) (B) were injected into the right inguinal mammary fat pad of female SCID, NOD-SCID or NSG mice (2×10^6^ cells). Tumour growth was monitored twice weekly and the values for all 8 mice were plotted to show the amount of variation in tumour volume at each time point. Line represents the median value.

When the average tumour volume was plotted against time, the overall tumour growth rate appeared to be similar across the different strains of mice. However, when the individual tumour volumes for each of the 8 mice (within a group) are plotted at each time point, a degree of variation can be seen. The variation in tumour sizes was greatest in NOD-SCID and SCID mice than in NSG mice, which showed a more uniform pattern of tumour growth for all cell lines tested (Figure 2 and data not shown). Figure 2A shows the variation in tumour growth of the parental MDA-MB-231 cell line in each of the three mouse strains. Each point represents one mouse and shows a tighter clustering of points in the NSG mice and a wider spread of points in NOD-SCID and SCID mice. Interestingly, the variation in individual tumour volumes within a group was increased following luciferase-tagging of the parental cell line (compared to the untagged parental cell line) in all three mouse strains, indicating that luciferase expression can alter the growth characteristics of tumour cells *in vivo* (Figure 2B) but not *in vitro* (data not shown).

### Analysis of Metastatic Spread and Survival after Primary Tumour Resection

**Figure 3:**
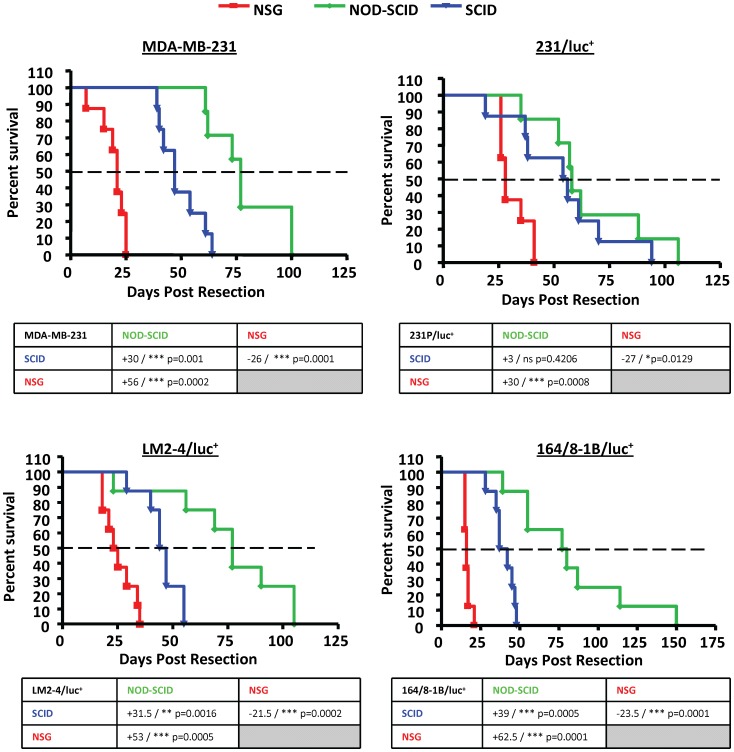
Survival of SCID, NOD-SCID and NSG mice following resection of the primary tumour. Metastatic spread and survival were monitored after resection of the primary tumour. Each graph shows the differences in survival of SCID, NOD-SCID and NSG mice for each cell line. Numbers represent the difference in median survival from the median survival of SCID mice (first row) and NSG mice (second row). Dashed line shows 50% survival. Statistical analysis was assessed by ANOVA.

**Figure 4:**
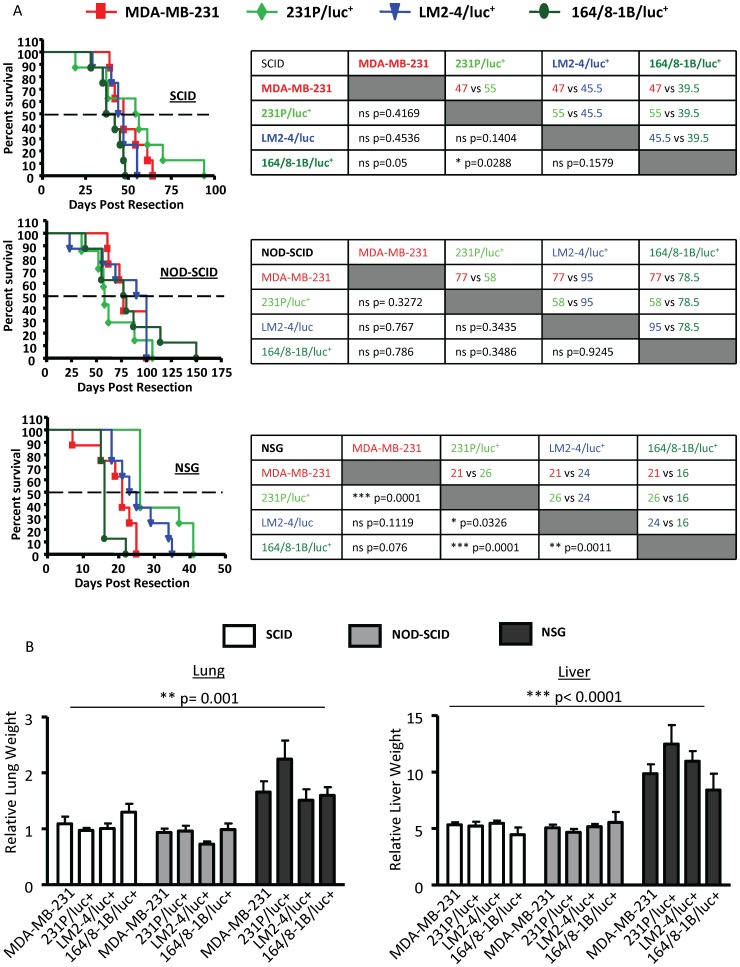
Comparison of survival of SCID, NOD-SCID and NSG mice after primary tumour resection. Following resection of the primary tumour, metastatic spread and survival were monitored. (A) Comparison of survival of each mouse strain after removal of MDA-MB-231, 231P/luc^+^, LM2-4/luc^+^ or 164/8-1B/luc^+^ primary tumours. Dashed line shows 50% survival. Numbers represent median survival and significance (P<0.05) was assessed by ANOVA. (B) Upon sacrifice, the lung and liver weights were recorded and calculated as a percentage of the total body weight. The relative lung and liver weights of NSG mice were significantly higher than those of NOD-SCID and SCID mice (**p = 0.001 and ***p<0.0001, respectively). Statistical analysis was assessed by ANOVA.

There was a marked difference in metastatic spread of disease, aggressiveness and survival following resection of the primary tumour between the different strains of mice, despite the fact that primary tumours grew at similar rates. The spread and progression of metastasis were much more aggressive in the NSG mice, leading to a worse survival outcome compared to either NOD-SCID or SCID mice (Figures 3 & 4). The median survival for the various cell lines ranged from 16–24 days in NSG mice, 39.5–55 days in SCID mice and 58–95 days in NOD-SCID mice. Contrary to expectation, NOD-SCID mice survived longer than SCID mice for all cell lines tested despite being more immune deficient than SCID mice.

When survival for each of the cell lines is compared within a strain, the maximum survival of any given cell line in NSG mice is approximately 40 days post resection, which is less than half the maximum survival of SCID and NOD-SCID mice (100 days and 150 days post resection, respectively) (Figure 4A). We did not find that the more metastatic variants (LM2-4/luc^+^ and 164/8-1B/luc^+^) caused a reduction in overall survival when compared to the parental cell line (MDA-MB-231) in any of the mouse strains tested. Since the luciferase-tagged parental cell line shows a prolonged survival in SCID and NSG mice, it is possible that luciferase expression may render cells less aggressive/metastatic.

The advanced metastatic disease in NSG mice included visceral metastases to many organs including the lung, liver, lymph nodes, heart, kidney, spleen, ovary and intestines (Table 1). In contrast, visceral metastases in SCID mice involved lung, lymph nodes, kidney, spleen and intestines, whereas, metastases in NOD-SCID mice primarily affected lymph nodes with few lung metastases (Table 1). We did not observe any evidence of brain metastases in these studies with the exception of one incidence (MDA-MB-231 in NSG mice), even though 164/8-1B/luc^+^ cell line was generated from a brain metastasis. However, it is of interest that 50% of the NSG mice implanted with the 164/8-1B/luc^+^ cell line had to be sacrificed due to ataxia, though evidence of brain metastases or other discernable cause for this could not be detected at necropsy. Furthermore, we observed that the parental MDA-MB-231 cell line was more aggressive in the NSG mice whereas it remains poorly aggressive in the SCID and NOD-SCID mice, giving rise to far fewer metastases, in terms of organs affected and metastatic burden (Table 1).

**Table 1:** Analyses of the metastatic spread of the various cell lines in SCID, NOD-SCID and NSG mice.

SCID	Regrowth	Lung	Liver	LN	Other	Ascites
**MDA-MB-231**	3/8 (37.5%)+++	6/8 (75.0%)+	0/8	3/8 (37.5%)+	2/8 (25.0%)+	0/8
**231P/luc^+^**	7/8 (87.5%)+++	6/8 (75.0%)+	0/8	4/8 (50.0%)++	4/8 (50.0%)++	2/8 (25.0%)+++
**LM2-4/luc^+^**	3/8 (37.5%)+++	6/8 (75.0%)++	0/8	5/8 (62.5%)+	4/8 (50.0%)++	0/8
**164/8-1B/luc^+^**	4/8 (50.0%)++	8/8 (100%)++	0/8	5/8 (62.5%)+	5/8 (62.5%)++	0/8
**NOD-SCID**	**Regrowth**	**Lung**	**Liver**	**LN**	**Other**	**Ascites**
**MMDA-MB-231**	4/8 (50.0%)+++	0/8	0/8	4/8 (50.0%)++	1/8 (12.5%)+++	1/8 (12.5%)++
**231P/luc^+^**	5/8 (62.5%)++	0/8	0/8	5/8 (62.5%)++	5/8 (62.5%)++	2/8 (25.0%)+++
**LM2-4/luc^+^**	2/8 (25.0%)+++	1/8 (12.5%)++	0/8	2/8 (25.0%)+	2/8 (25.0%)+	1/8 (12.5%)+++
**164/8-1B/luc^+^**	6/8 (75.0%)+++	0/8	0/8	6/8 (75.0%)++	5/8 (62.5%)+++	3/8 (37.5%)+++
**NSG**	**Regrowth**	**Lung**	**Liver**	**LN**	**Other**	**Ascites**
**MDA-MB-231**	1/8 (12.5%)+++	8/8 (100%)+++	8/8 (100%)+++	5/8 (62.5%)++	*4/8 (50.0%)++	0/8
**231P/luc^+^**	6/8 (75.0%)+++	8/8 (100%)+++	8/8 (100%)+++	8/8 (100%)++	7/8 (87.5%)+++	5/8 (62.5%)+++
**LM2-4/luc^+^**	5/8 (62.5%)++	8/8 (100%)+++	8/8 (100%)+++	7/8 (87.5%)++	5/8 (62.5%)+	8/8 (100%)+++
**164/8-1B/luc^+^**	6/8 (75.0%)+	8/8 (100%)+++	8/8 (100%)+++	7/8 (87.5%)+++	8/8 (100%)+++	2/8 (25.0%)+

Numbers of mice out of 8 mice that have tumours in different organs; shown as percentages in parentheses. LN- lymph nodes; MFP- mammary fat pad; other- visceral metastases to various organs including spleen, kidney, ovary, heart, brain, intestines. Brain metastasis was only detected in one mouse. The occurrence of ascites generally resulted in formation of intestinal metastases.+++Large regrowth (often ulcerated) or extensive metastatic burden in liver/lung or involving 3 or more LNs/other organs.++Intermediate sized regrowth or moderate liver/lung metastases or involving 2 LNs/other organs.+Small regrowth or minimal liver/lung metastases or involving only one LN or other organ.

LN - lymph node; other - visceral metastases to different organs including kidney, heart, spleen, ovary, brain, intestines. *Only 1 brain met was detected. Ascites often resulted in intestinal metastases.+++Large regrowth (often ulcerated) or extensive metastatic burden in liver/lung or involving 3 or more LNs/other organs.++Intermediate sized regrowth or moderate liver/lung metastases or involving 2 LNs/other organs.+Small regrowth or minimal liver/lung metastases or involving only one LN or other organ.

Consistent with an increased metastatic burden in NSG mice, the liver and lungs of these mice were completely overrun (though not enlarged) with numerous large tumour nodules, resulting in increased organ weights in these mice compared to NOD-SCID and SCID mice (Figure 4B). Since the size of nodules varied considerably between the strains and the numbers of nodules were often too many to count, we expressed metastatic burden as organ weight (liver or lung) relative to final weight of the mouse. A liver preference in NSG mice has previously been reported in several studies [12], [15], [16]. The development of ascites in NSG mice was most likely due to liver failure resulting from the extensive liver nodules that were predominantly observed in this strain, 47% of NSG mice (compared to 6% of SCID mice and 22% of NOD-SCID mice) and often resulted in the formation of metastases in the intestines (Table 1).

As mentioned previously, tumour resection was necessary to allow the mice to survive long enough for the establishment of extensive metastases. In NOD-SCID or SCID mice some metastases are detected 1–3 weeks post resection, although overt multi-organ metastasis is not detected until 5–8 weeks post-resection (data not shown). This is in stark contrast to NSG mice where overt metastases are detected 1–2 weeks post resection (data not shown). Tumour regrowth, determined as tumour recurrence at or near the implantation site, is often observed and reduces the duration of the experiment particularly when tumour regrowth volumes reach institutional endpoint criteria before visceral metastases have become established. Although tumour regrowth was observed in all three strains of mice, it did not seem to impede the metastatic spread of disease in NSG mice, as was the case in NOD-SCID and SCID mice, where the size of the regrowth was a common reason for sacrifice. While tumour resection appears not to be necessary in order to detect metastases in NSG mice [24], it is necessary to generate spontaneous metastases consistently in NOD-SCID and SCID mice [1].

### Effects of Luciferase-tagging MDA-MB-231 on Tumour Growth and Metastatic Spread

**Figure 5:**
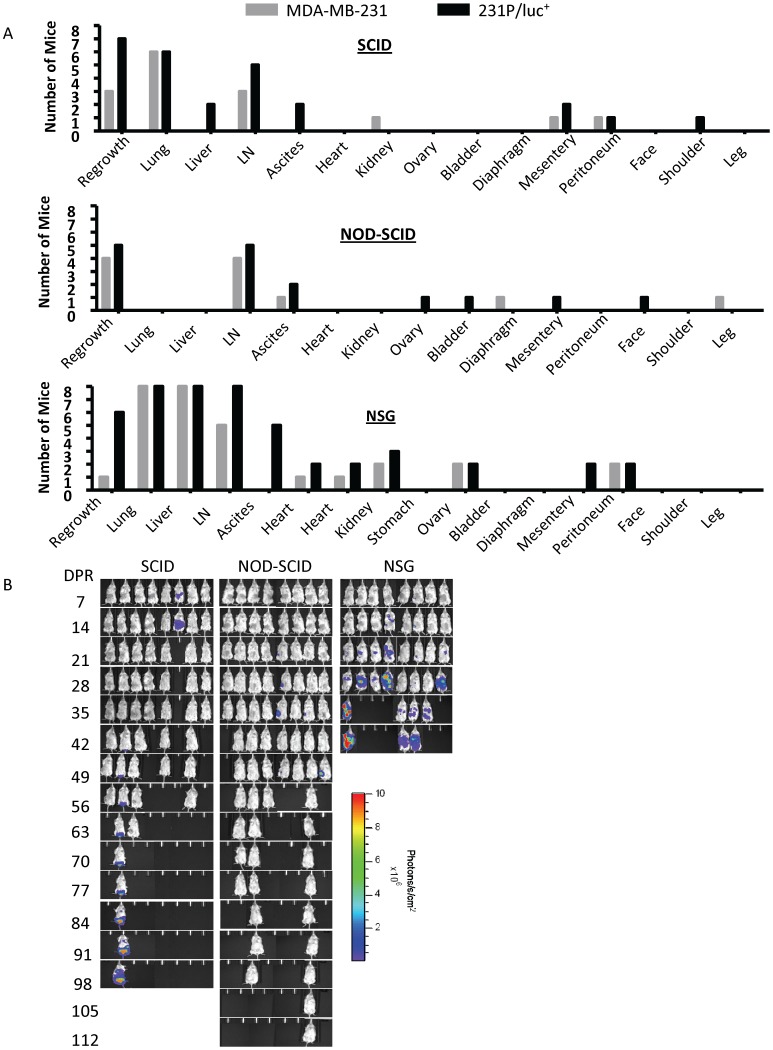
The effect of luciferase on metastatic burden in SCID, NOD-SCID and NSG mice. (A) Histograms showing the number of mice with metastases in various organs following implantation and resection of the parental MDA-MB-231 cell line or the luciferase-tagged parental cell line (231P/luc^+^). A greater metastatic burden was observed in NSG mice compared to either SCID or NOD-SCID mice, in which multiple organs were affected. (B) Whole body bioluminescence images of the emerging metastases in SCID, NOD-SCID and NSG mice following resection of a primary orthotopic tumour generated by implantation of the 231P/luc^+^ cell line.

The ability to monitor metastatic spread using non-invasive whole body bioluminescence greatly facilitates the ability to use our models to study advanced metastasis. However, during the course of our studies, we observed several unexpected consequences following luciferase-tagging of the parental cell line. Whilst some of these changes are subtle, such as an increase in tumour volume variation within a group (Figure 2), others are more overt, such as the loss of luciferase signal and a change in the pattern of metastatic disease (Figure 5). It is possible that the degree of these alterations may be linked in part to the immune/para-immune status of the host animal since we found that in NSG mice, the parental cell line and the luciferase-tagged parental cell line gave rise to a similar overall spectrum of organs/tissues affected by metastatic disease (i.e., lung, liver, lymph node, heart, kidney, ovary, peritoneum), which is in contrast to both NOD-SCID (lymph nodes only) and SCID mice (lung, lymph nodes, mesentery, peritoneum; Figure 5A) where the spread of disease showed more variation.

Whilst the luciferase signal was maintained in NSG mice for the duration of the experiment (42 days following resection of the primary tumour), we found that in the SCID and NOD-SCID mice the luciferase signal was lost over time. There was little or no whole body bioluminescence signal even though all of the mice had metastatic disease and in some cases primary regrowth in spite of a negative whole body bioluminescence signal (Figure 5B and Table 2). We did not experience a loss of luciferase signal with our other luciferase-tagged cell lines, LM2-4/luc^+^ or 164/8-1B/luc^+^.

**Table 2:** Difference in sacrifice endpoints of mice with MDA-MB-231 or 231P/luc^+^ metastatic disease – comparison between SCID, NOD-SCID and NSG mice.

	SCID		NOD-SCID		NSG	
REASON FOR SACRIFICE	MDA-MB-231	231P/luc^+^	MDA-MB-231	231P/luc^+^	MDA-MB-231	231P/luc^+^
Hunched posture, ruffled fur,lethargy and/or laboured breathing	3/8	4/8	2/7	3/7	4/8	2/8
Large regrowth (institutional endpoint)	2/8	2/8	3/7	2/7		1/8
Hind limb paralysis					4/8 cause unknown	3/8 cause unknown
Forelimb paralysis	1/8			1/7 axillary LN tumour		2/8 axillary LN tumour
Other	2/8 found dead	1/8 large tumour LNbetween shoulders; 1/8 lacerated tail thatwasn’t healing	2/7 experiment terminated – no signof metastasis	1/7 large facial LNtumour preventingeating & drinking		

Despite the fact that few of the SCID or NOD-SCID mice showed signs of luciferase-positive metastatic disease by bioluminescence, tumours were identified at necropsy in all mice.

## Discussion

The main objective of these studies was to compare rates and patterns of spontaneous metastases in SCID, NOD-SCID and NSG mice following orthotopic implantation of human breast cancer cells and resection of the primary tumour. Although several investigators have evaluated primary tumour growth efficiency following titration of various types of cancer cells [8], [16] and human tissue samples from patients [14], very few studies have been dedicated to the analysis of metastases in these strains. Moreover, of the studies that have evaluated metastasis, few involved the use of orthotopic implantation methods [24]; the majority of studies relied upon ‘artificial’ metastasis models, such as direct intravenous cell injection [9], [12] or intra-splenic tumour cell injection followed by removal of the spleen [16]. However, we have recently reported that intrasplenic tumour cell injection results in colonization of tumour cells in the liver, just one day after cell injection. Consequently, the rapid localization of cancer cells to the liver can only be explained by embolization, and as such precludes the development of authentic spontaneous metastases [6]. As far as we are aware, this is the first study to compare metastasis in different strains of immune-suppressed mice following orthotopic implantation of human cancer cells and resection of the primary tumour - a scenario akin to the surgical removal of primary breast cancers in patients.

As mentioned previously, reducing the host immunity has been shown to correlate with an improved tumour take; a superior tumour take in NSG mice, compared to NOD-SCID and SCID mice, has been demonstrated when using limiting dilutions of cells in both primary tumour and experimental metastases studies. However, since the major aspect and purpose of our study was to analyse the differences in *spontaneous* metastases in these different strains of mice following primary orthotopic tumour resection, we injected a high number of cells into the mammary fat pads of mice to ensure consistent and comparable primary tumour growth in all mice. Surprisingly, this resulted in similar primary tumour growth rate patterns across the three mouse strains, regardless of the cell line used. However, in spite of the observed similarity in primary tumour growth, we observed striking differences in metastatic aggressiveness and survival in the various strains of mice. Thus, we found that primary tumour growth rate alone was unable to predict disease outcome but rather that metastatic aggressiveness could do so, and this was related to differences in host immune characteristics and status.

An immune component to cancer progression has long been recognized following the identification of leucocytes in tumours by Rudolf Virchow in the 19^th^ century, but it is only recently that the critical role of this complex relationship has been uncovered. In order for tumours to arise, tumour cells must evade early immune surveillance mechanisms and perturb host immunity, thereby creating a ‘tumour-promoting’ inflammatory environment [25], [26]. The tumour microenvironment contains many innate and adaptive immune cells [27], in addition to cancer cells and their surrounding stroma, which together play key roles at various stages of tumour development and progression including tumour initiation, growth, invasion and metastasis [28]. The latter requires cells to acquire a more motile fibroblast characteristic so that they can invade epithelial linings and basal membranes to reach the underlying blood or lymphatic vessels. Once cancer cells have intravasated into vessels, they interact with various circulating cells in order to survive the travel through vessels until they become arrested in the capillary bed and extravasate into tissues, where the metastatic progenitors interact with immune, inflammatory and stromal cells and start to proliferate [29]. In some cases, cells may be targeted to a premetastatic niche in response to tumour-generated inflammatory signals prior to the arrival of the metastasis-initiating cancer cells [30]. However, only a minority subset of cancer cells (e.g., 0.01%) that enter the circulation will actually survive to give rise to micrometastases [31].

Our results indicate that the immune status of the host can hugely impact the sites affected by metastasis and the severity of metastatic disease progression. Human tumours can only successfully engraft in mice with an impaired immune system, therefore, many immune-deficient models have been developed for the purposes of studying human tumour biology and subsequent anti-tumour therapies. Severe combined immunodeficient (SCID) mice are commonly used as recipients of human cells because they lack adaptive immunity (B and T cells). A more immune deficient NOD-SCID strain was generated when the *scid* mutation was introduced into the non-obese diabetic (NOD) background, resulting in mice with *reduced* macrophage and natural killer (NK) cell function, absent complement-dependent hemolytic activity [32], [33] and an improved tumour engraftment. However, in our study, we did not observe a more aggressive metastatic spread or worse survival outcome in NOD-SCID mice compared to SCID mice, as would be expected based on host immunity. We speculate that this might be due to the *variable* residual NK cell activity and macrophage function retained in these mice, and which likely affects the metastatic process. We suspect that this residual immunity gives rise to the variable metastatic spread observed in SCID and NOD-SCID mice in contrast to the uniform metastatic spread in NSG mice. It is possible that the variability exhibited by NOD-SCID and SCID mice may be exacerbated by primary tumour regrowth since this not only reduces the duration of the experiment but the presence of a primary tumour may suppress the growths of metastases [34].

We found that the time required for metastasis to occur was greatly prolonged in NOD-SCID and SCID mice compared to NSG mice and was more variable. Studies showing that further immunocompromising mice using whole body irradiation, antibodies targeting NK cells, genetic manipulation to decrease immunity (e.g. NOD-SCID-*β2m*-deficient or NOD-SCID-*IL-2R*γ-deficient mice) leads to more effective tumour growth [12]. The NOD-SCID-*IL-2R*γ (NOG or NSG) deficient strain lack the interleukin 2 receptor gamma chain (*IL-2R*γ) which is shared by several interleukin receptors (i.e. receptors for IL-2, IL-4, IL-7, IL-9, IL-15 and IL-21) [35] and results in an absence of functional NK cells which require IL-15 signalling to develop [35]. Our studies show that spontaneous multi-step metastatic spread is accelerated and survival is markedly reduced in the highly immunocompromised NSG mice when compared to both NOD-SCID and SCID mice. These findings suggest that the differences between NSG mice and NOD-SCID mice have an enormous impact on metastasis, these mainly being NK cell function and interleukin 2 signalling. However, the differences in engraftment and metastatic spread between NSG and NOD-SCID mice are likely caused by immunological (signalling) factors rather than immune cell deficiencies (T-cell, B-cell, macrophage, NK cell) [13] since only minor engraftment differences have been reported in NOD-SCID mice with and without functional NK cells [13].

Overall, these differences highlight the importance of immunity in the spread of metastatic disease. NSG mice are extremely immunocompromised and therefore, provide a permissive environment for metastasis after primary tumour resection. This intricate relationship between cancer progression, immunity and inflammation can also affect responses to therapy, since induction of tumour cell death and presentation of tumour antigens can elicit an immunogenic response that in turn activates an immune response [36]. In this way, the response to chemotherapy and/or radiotherapy may combine both direct cytotoxic effects with the development of long-term anti-tumour immunity [37], which would likely be altered in hosts with reduced immune status.

Our prior studies of spontaneous metastases have been greatly enhanced by the ability to monitor, the spread of disease non-invasively over prolonged time periods, using whole body bioluminescence [38]–[40]. However, we have found that modifying cell lines to express luciferase can alter cellular properties in a manner that may not always be immediately apparent. For example, in this study, we compared the untagged and luciferase-tagged MDA-MB-231 cell line and found an increased variation in tumour growth within a group and altered metastatic spread in the luciferase expressing cells. This is not the first time that we have noted a difference in the behavior of luciferase-tagged and untagged cells; we have observed a slight reduction in metastatic aggressiveness when the untagged lung metastatic variant (LM2-4) is compared with the luciferase-tagged metastatic variant (LM2-4/luc^+^). More specifically, while the untagged LM2-4 variant reliably gives rise to visceral metastases in lungs, livers and lymph nodes of SCID mice, the luciferase-tagged variant results in lung and lymph node metastases but seldom generates liver metastases in these mice ([3] & unpublished observations). However, a reduction in metastatic aggressiveness of the LM2-4/luc^+^ cell line was not apparent in NSG mice where it consistently gave rise to lung, liver and lymph node metastases.

The overall selection pressures of cancer cells in culture tend to make them more homogeneous, a fact that is highlighted by the selection and expansion of luciferase positive clones. It is currently not clear whether the reduced metastatic ability of luciferase-tagged cells is a consequence of luciferase expression *per se* or whether it simply reflects the varying metastatic potential of the different cells selected during the *in vitro* cloning process. The LM2-4 and 164/8-1B metastatic variants were initially selected on the basis of their *in vivo* aggressiveness prior to being transfected with luciferase. Therefore, any loss in metastatic ability in these cell lines may be minimal (and not easily detected) since they are already highly metastatic *in vivo*. This is in contrast to the luciferase-tagged parental MDA-MB-231 cell line, which was solely selected on the basis of *in vitro* luciferase expression and likely consists of cells with varying degrees of metastatic potential. The luciferase-tagged parental cell line and the LM2-4/luc^+^ were shown to express luciferase with similar intensities *in vitro*. However, it has been reported that high (but not low) luciferase expression in an ovarian cancer cell line can severely impair tumour growth *in vivo*, though this was linked to repeated luciferin injections over time [41].

Not only have we observed a reduction in metastatic aggressiveness in cells expressing luciferase but we have also noted tumours devoid of a bioluminescence signal in mice initially implanted with luciferase-tagged parental cells. Whether this simply signifies a loss of bioluminescence signal over the course of disease or whether this is the result of an active *in vivo* selection pressure, for example the silencing of luciferase expression by DNA methylation is currently unknown (personal communication, Othon Iliopoulous to R.S.K). These findings may suggest another factor, in addition to hypoxia and tumour necrosis [42], [43] that reduces the ability of bioluminescent imaging to precisely predict disease burden and response to therapy and should be considered in any investigation involving luciferase-tagged cell lines.

Taken together, our data are the first to show that host immune or para-immune defences can significantly impact the spread of spontaneous visceral metastases and survival following resection of a primary orthotopic (breast) cancer in circumstances where the growth of primary tumours is not necessarily similarly affected. Elucidation of the additional host cellular defects in NSG mice compared to SCID and NOD-SCID mice could reveal promising therapeutic strategies that are effective at preventing the progression of metastatic disease or in treating overt metastatic disease.
